# Marijuana Use Is Associated with Behavioral Approach and Depressive Symptoms in Adolescents and Emerging Adults

**DOI:** 10.1371/journal.pone.0166005

**Published:** 2016-11-11

**Authors:** Natasha E. Wright, Danny Scerpella, Krista M. Lisdahl

**Affiliations:** Department of Psychology, University of Wisconsin-Milwaukee, Milwaukee, WI, United States of America; Chiba Daigaku, JAPAN

## Abstract

**Background:**

Repeated CB1 binding due to THC results in downregulation of the endocannabinoid system in cortex and limbic regions, perhaps disrupting frontolimbic functioning. This is particularly a concern in young adults who are still undergoing neurodevelopment in frontal and limbic regions. Such disruptions may be linked to increased depressive symptoms, anxiety symptoms, and executive dysfunction, and decreased behavioral approach.

**Objectives:**

Here we examine the influence of young adult marijuana use on anxiety, depressive symptoms, behavioral approach, and executive dysfunction. The influence of alcohol and gender were also assessed.

**Methods:**

84 participants (42 MJ, 42 controls) aged 18–25 were balanced for gender (39 F). Exclusion criteria included: MRI contraindications, left handed, comorbid Axis-I disorders, major medical or neurologic disorders, prenatal issues, or prenatal alcohol/illicit drug exposure, or excessive other drug use. Participants completed the FrsBE, BIS/BAS, State-Trait Anxiety Inventory (State), and BDI-II. Multiple regressions were run to predict anxiety, depressive symptoms, behavioral approach, and executive dysfunction from MJ group status, past year alcohol use, gender, and MJ*gender interactions, controlling for cotinine and ecstasy.

**Results:**

MJ group predicted increased depressive symptoms (p =.049). Decreased fun-seeking (p =.04), reward response (p =.01), and BAS total (p =.01) were predicted by MJ group. Gender predicted decreased reward responsiveness in females (p =.049) and decreased BIS in females (p =.03). Female marijuana users had increased anxiety symptoms (p =.04) and increased disinhibition (p =.04). Increased cotinine predicted increased drive (p =.046), reward responsiveness (p =.008) and BAS Total (p =.02). Apathy and Executive Dysfunction were not predicted by any measures. All results had small effect sizes.

**Conclusions/Importance:**

Depressive symptoms were greater in MJ users, while behavioral approach was decreased. Cotinine levels predicted increased behavioral approach. Female MJ users also had greater anxiety and disinhibition. In sum, these findings suggest sub-clinical threshold deficits related to regular marijuana use that are indicative of a need to prevent marijuana use in adolescents and young adults.

## Introduction

As the most commonly used illicit drug, marijuana is used by 44.4% of 12th graders and over 57.5% of young adults in their lifetime [1, 2]. In general, individuals begin using marijuana during adolescence, and usage peaks between the ages of 18–25 [3]. Chronic marijuana exposure may result in greater neurocognitive deficits and mood symptoms in this population due to ongoing neurodevelopment occurring throughout adolescence and emerging adulthood[4]. A decline in the perceived risks associated with marijuana use[5] combined with a recent (past ten years) doubling of past year marijuana use among young adults (aged 18–29; [6]) emphasizes the importance of understanding the neurocognitive and mood consequences of regular marijuana use in youth.

The endocannabinoid (eCB) system plays a key neurodevelopmental role [7, 8]). The primary brain cannabinoid receptor, CB1, has significantly greater binding in adolescence than in adulthood [9], and is activated by delta^9^-tetrahydrocannabinol (THC), the major psychoactive component of marijuana. This binding modulates the reward system within the ventral tegmental area (VTA) of the brain, increasing the release of dopamine[10]. Repeated CB1 binding due to exogenous cannabis (THC) exposure results in downregulation of the eCB system [11], particularly in limbic regions, such as the hippocampus (see [12]).

The endogenous eCB system has been implicated in mood symptomatology as well as in executive functioning deficits [13–15], perhaps due to its concentration of CB1 receptors in prefrontal and limbic regions [16]. As frontal and limbic neuroanatomy changes during adolescence and into young adulthood [17], so too does the eCB system [7, 18]. It is unsurprising, then, that marijuana use specifically has been implicated in a rise in executive dysfunction, anxiety, depressive symptoms, and increased impulsivity in adolescents and emerging adults in particular [4].

In this sensitive and dynamic neurodevelopmental time period [17], regular (weekly to daily) marijuana use has been found to have significant neural and functional impact. These include several neurocognitive deficits, including in attention [19–22], executive functioning [23–25], and impulsive behavior and inhibition [23, 26–30]. Structural imaging studies have primarily found abnormalities in frontolimbic regions [31–42]. Given these frontolimbic abnormalities, it is important to consider the impact of chronic marijuana exposure on mood and self-reported symptoms of executive dysfunction.

Mood symptoms in the absence of an Axis-I disorder and day-to-day deficits in everyday executive functioning, as exhibited through behavioral deficits, have been relatively overlooked in the marijuana literature. This is an important consideration given that traditional neuropsychological measures may not capture every-day dysfunction in substance using populations [43]. In a longitudinal study by Felton and colleagues [44], self-reported and behavioral measures of disinhibition in 8^th^ grade prospectively predicted increased marijuana use across high school students, regardless of gender. Self-reported apathy and executive dysfunction on the Frontal Systems Behavioral Scale (FrSBe) have also been related to severity of marijuana use [45]. The most extensive evidence of everyday behavioral deficits comes from self-reported impulsivity in marijuana users, as measured by the Barratt Impulsivity Scale (BIS-11) with facets of motor function, nonplanning, and attention [27, 37, 46–50]. However, the behavioral approach and behavioral inhibition aspects of impulsivity have, to our knowledge, only been investigated in one study, which found no relationship between regular marijuana use and behavioral approach scores [51]. In investigating mood symptoms and executive dysfunction, our group has previously reported that in young adult polydrug users, marijuana use significantly predicted anxiety and depressive symptoms, while past year alcohol use predicted executive dysfunction and disinhibition [52]. Consistent with these findings, several other studies also report increased risk of depressive and anxiety symptoms in regular marijuana users (for review, see [53]). However, not all studies have found a relationship between marijuana use and mood (e.g., [23, 28]) or cognitive deficits (e.g., [54]). As there has been limited research into other facets of impulsivity, such as behavioral approach and avoidance, and particularly limited investigation into self-reported executive dysfunction, more research is needed in these areas. Further, as gender and alcohol use are both known to influence impulsivity, mood, and executive functioning, assessment of their potential moderating impact is needed.

Many of these findings may not be unique to marijuana users. Multiple studies have found similar executive dysfunction and psychological symptomatology increases in youth with alcohol use disorders and binge drinking histories (see [55]). Marijuana and alcohol are also often used together simultaneously [56], and both THC and alcohol regulate eCB signaling. While THC binds directly with CB1 receptors, alcohol interacts indirectly through GABAergic and glutamatergic neurons [57, 58]. Similar to THC, regular alcohol use also leads to downregulation of CB1 receptors [58]. As both substances act within the eCB system, it is important to take into consideration how either may be independently affecting function. Therefore, the current study examines the influence of both alcohol and marijuana use on mood and executive function symptoms.

Finally, an often-important moderator of substance-related psychological symptomatology and executive functioning is gender. Female marijuana users with a lifetime cannabis use disorder tend to have higher incidence of anxiety or mood disorders than male users with a cannabis use disorder [59], as is also true in the general population [60]. Prefrontal and amygdala volumes have also been found to differ by gender in marijuana users, with females exhibiting greater abnormalities which were linked with executive dysfunction and increased depressive symptoms [39, 41]. One reason for the potential additional susceptibility to the neurotoxic effects of marijuana may be due to altered signaling in the CB1 receptor in females, but not males [61, 62]. More widespread effects of THC in adolescent female rats than in male rats have also been observed [63]. Therefore, when examining consequences of marijuana exposure, it is important to examine potential gender differences.

The present study examined the influence of marijuana use on anxiety, depression, impulsivity, and executive dysfunction. We hypothesized that marijuana use will be predictive of greater dysfunction and symptoms than healthy controls. Specifically, we predicted that marijuana users would exhibited higher levels of anxiety, depressive symptoms, and executive dysfunction, as well as facets of impulsivity characterized as increased behavioral approach and decreased behavioral avoidance. As marijuana users often also engage in heavy drinking and as gender may moderate the effects of marijuana use, the influence of both alcohol and gender was assessed. It was hypothesized that greater alcohol use would similarly be associated with increased mood symptoms and decreased executive functioning. Further, it was hypothesized that gender would influence functioning, such that female users would experience greater symptoms of functional impairment than male users.

## Materials and Methods

### Participants

Eighty-four participants (42 MJ users, 42 controls) were recruited through local newspaper advertisements and fliers placed around campus at the University of Cincinnati. Groups were balanced for gender (MJ: 16 female; Controls: 23 female). Inclusion criteria included being a fluent English speaker 18–25 years old. Participants were considered MJ users if they had smoked more than 10 times in the past year or more than 500 times lifetime (i.e., either being a current user or previous heavy user), and had less than 10 other drug uses. Healthy controls had smoked MJ less than 5 times past year and less than 20 times lifetime. Exclusion criteria for both groups included: being left handed, MRI contraindications, lifetime history of an independent Axis-I disorders according to DSM-IV criteria (i.e., the symptoms are not due to marijuana use), major medical or neurologic disorders, prenatal issues (e.g., gestation <35 weeks) or prenatal alcohol (>4 drinks/day or >7 drinks/week) or illicit drug (>10 uses) exposure, or excessive drug use in lifetime (>10 uses of any drug category except nicotine, alcohol, or marijuana). Participants were required to remain abstinent from alcohol and drug use for seven days leading up to the study session, as confirmed through self-report and drug toxicology screen; this time period is typically a long enough period of time for the most substantial withdrawal symptoms to subside [64].

### Procedure

Participants were recruited for the parent imaging genetics study (PI: Lisdahl, 1R03 DA027457). Interested participants called in and were screened using a semi-structured phone interview for Axis-I disorders according to DSM-IV criteria. If still eligible, participants completed written informed consent and the study protocol in either one or two sessions. Those with substance use histories completed the psychological questionnaires, drug use interview, and neuropsychological battery in two sessions (typically 2–3 days apart). Those with minimal use completed the study in one session. Participants were paid $160 for two sessions ($110 for one) and received parking reimbursement, local substance treatment resources and images of their brain. The University of Cincinnati IRB approved all aspects of this study, including the consent procedure.

### Drug Use

Marijuana, alcohol, and other drug use were measured using a modified version of the Timeline Follow-Back (TLFB) [28, 65]. Utilizing memory cues of common holidays and personal events, participants recounted frequency of drug use over the past year (assessed month-by-month for one year). Additionally, a semi-structured interview was administered to measure frequency/quantity of lifetime drug use [28]. For each drug category, participants were asked their average weekly use for each year of use. The following drug categories were assessed: ecstasy or Molly, alcohol, marijuana, sedatives (e.g., downers, ketamine, GHB), stimulants (amphetamine, methamphetamine, cocaine, crack cocaine), hallucinogens (mushrooms, PCP, LSD, peyote), opioids (heroin, opium), and inhalants (nitrous oxide, paint, glue, household cleaners, gas). The participant’s drug use was measured in standard units (joints for marijuana; standard drinks for alcohol; tablets for ecstasy).

### Self-Report Symptom Inventories

Anxiety. The State-Trait Anxiety Inventory (STAI; [66]) State subscale measures temporary or “state” anxiety. Total raw STAI-state was used in the present analysis. Depression. Depression was measured by the Beck Depression Inventory—2^nd^ Edition (BDI; [67]). The BDI is a 21-item measure of depressive symptoms over the past 2 weeks. Total raw BDI score was used in the present analysis. Self-Reported Executive Functioning. Executive functioning in day-to-day life was measured by the 46-item Frontal Systems Behavioral Scale (FrSBe; [68]). As this measure was designed to assess daily functioning before and after an acute illness or neurological illness, participants only filled out the “after” portion to report on their current levels of functioning. Impulsivity/Behavioral Approach. The Behavioral Inhibition System and Behavioral Approach System (BIS/BAS; [69]) is a 30-item inventory measuring approach and avoidance behaviors of moving towards or away from appetitive or unpleasant stimuli, respectively, with increased BAS and decreased BIS scores being related to increased impulsivity and response to reward cues, while decreased BAS may be indicative of more apathy and decreased response to positive reinforcement. The subscales that make up the BAS include reward responsiveness (anticipation of reward), fun seeking (desire for new and novel positive reinforcers), drive (pursuit of goals), and BAS total (overall happiness and experience of a positive reinforcer). The BIS does not have subscales, but is one overall score that, when higher, reflects generally more anxious and punishment-responsive personality traits.

### Data analysis

Between-group differences on demographic variables were measured with analyses of variance (ANOVAs) and χ2 tests; no significant differences between groups emerged, and therefore demographic variables were not included in subsequent analyses as covariates. The one exception was in gender, which, while it did not differ by groups, was included a priori in the study aims. All variables were normally distributed, except depressive symptoms and reward response. These two variables were therefore log transformed to increase normality of distribution. Urine cotinine (NicAlert^™^, JANT Pharmacal Corporation, Encino, CA, USA), a metabolite of nicotine, was included in all analyses as a covariate, as nicotine withdrawal has been shown to alter neurocognitive functioning in adolescents in general [70] and in marijuana users in particular [71], and as past year cigarette use differed by groups. Ecstasy was similarly included as a covariate, as groups differed in past year ecstasy use. After controlling for potential confounds (i.e., cotinine level, ecstasy), multiple regressions were run to examine whether marijuana (MJ) group status or alcohol use independently predicted mood or functioning symptoms. The potential interactive effect of MJ group and gender was also assessed as a second block in the regression. All interpretations of statistical significance were made if p <.05. Due to power issues, there was no correction for multiple comparisons.

## Results

### Demographics

Groups did not differ significantly on age [F(1,82) =.04, p =.85], education [F(1,82) = 2.72, p =.10], reading level (from the WRAT-IV) [F(1,82) = 1.25, p =.27], gender (1 = male, 2 = female) [x^2^ = 2.35, p =.13], race (1 = not Hispanic or Latino; 2 = Hispanic or Latino; 3 = unknown) [x^2^ = 1.05, p =.31], or ethnicity (1 = American Indian; 2 = Asian; 3 = Native Hawaiian/Pacific Islander; 4 = Black/African American; 5 = White/Caucasian; 6 = more than one ethnicity) [x^2^ = 3.34, p =.50]. See Table 1.

**Table 1:** Demographics by Group.

	Controls (n = 42) % or *M* (SD)+ Range	MJ (n = 42) % or *M* (SD)+ Range
Age	21.1 (2.2) 18–25	21.2 (2.4) 18–25
Education	13.9 (1.7) 11–18	13.3 (1.8) 9–17
Reading Score (WRAT-IV)	100 (10.5) 78–120	103 (15.1) 73–134
Gender (% female)	55%	38%
% Caucasian	60%	67%
*Past Year Marijuana Use (joints)	0.1 (.4) 0–2	644 (1272.1) 0–7343
*Lifetime Marijuana Use (joints)	1.64 (3.7) 0–15	2483.3 (4937.4) 26–23838
*Past Year Alcohol Use (standard drinks)	68.2 (91.7) 0–459	330 (400.8) 0–7343
*Lifetime Alcohol Use (standard drinks)	329.93 (519.75) 0–2280	1358.5 (1544.9) 14–6191
*Past Year Ecstasy Use	0 (0) 0–0	.21 (.6) 0–3
*Cotinine Level	1.21 (2.0) 0–6	3.71 (2.4) 0–6

^+^M = mean; SD = standard deviation.

*p <.05

### Drug Use Patterns

As would be expected, groups different significantly in drug use patterns, such as cotinine level [F(1,82) = 26.67, p <.001], past year alcohol use [F(1,82) = 16.97, p <.001], past year marijuana use [F(1,82) = 10.76, p =.002], past year cigarette use [F(1,82) = 5.47, p =.02], and past year ecstasy use [F(1,82) = 4.63, p =.03].

### Clinical Elevations

We examined the percentage of controls and marijuana users who demonstrated clinical elevations on each of the anxiety, depressive and executive function scales (see Table 2), though it is important to note that no subjects met criteria for current or lifetime psychiatric independent diagnoses. Seven percent of marijuana users and 2% of controls reached clinical threshold for depressive symptoms (>14 raw score on the BDI-II; *x*^2^(1) =.37, *p* =.54). In the MJ group, one individual (2%) reached a clinical threshold of one SD above the mean for anxiety while, in the control group, 5% of healthy controls reached threshold (T-scores above 60; *x*^2^(1) =.40, *p* =.54). For self-reported executive functioning, elevations in symptoms were defined as a T-score above 65. Seventeen percent of marijuana users and 14% of controls were elevated in their apathy scores (*x*^2^(1) =.09, *p* =.76); 21% of marijuana users and 4% of controls were elevated in their disinhibition scores (*x*^2^(1) = 2.28, *p* =.13); and 36% of marijuana users and 14% of controls were elevated in their executive dysfunction (*x*^2^(1) = 3.94, *p* =.05).

**Table 2:** Clinical Levels of Symptom Scales.

	Marijuana Users				Controls			
	M	SD	Range	Elevated Score*	M	SD	Range	Elevated Score*
BDI-II	6.0	5.6	0–26	7%	4.7	6.7	0–41	2%
STAI-State	43.7	7.4	36–68	2%	42.5	7.3	34–64	5%
Apathy+	53.1	12.8	23–84	17%	53.1	15.0	25–106	14%
Disinhibition+	56.5	12.6	31–100	21%	50.7	12.6	28–84	4%
Exec Dysfunction+	57.9	13.0	21–81	36%	52.1	12.7	31–86	14%

^+^Apathy, Disinhibition, and Executive Dysfunction (Exec Dysfunction) are subscales from the Frontal Systems Behavior Scale (FrSBe); normative T-scores are presented.

*Elevated scores denote the percentage of individuals who demonstrated scores that were greater than 1 SD T-score above the normative group’s mean on the STAI, above 65 T-score on the FrSBe scales, and raw scores >14 on the BDI-II.

### Primary Analyses

#### Group Results

MJ group status significantly predicted increased **depressive** symptoms [beta =.30, t = 2.00, p =.049, f^2^ =.055]. On the BIS/BAS, **fun-seeking** was negatively predicted by MJ group [beta = -.30, t = -2.10, p =.04, f^2^ =.06]. Decreased **reward response** [beta = -.36, t = -2.55, p =.01, f^2^ =.09] and decreased **BAS System Total** were also predicted by MJ group status [beta = -.38, t = -2.60, p =.01, f^2^ =.09].

#### Gender Results

Gender predicted **reward response** with females having decreased reward responsivity [beta = -.22, t = -2.00, p =.049, f^2^ =.10]. **BIS System Total** was predicted by gender [beta = -.24, t = -2.18, p =.03, f^2^ =.065], with females having lower BIS total.

#### Group*Gender Results

**Fig 1:**
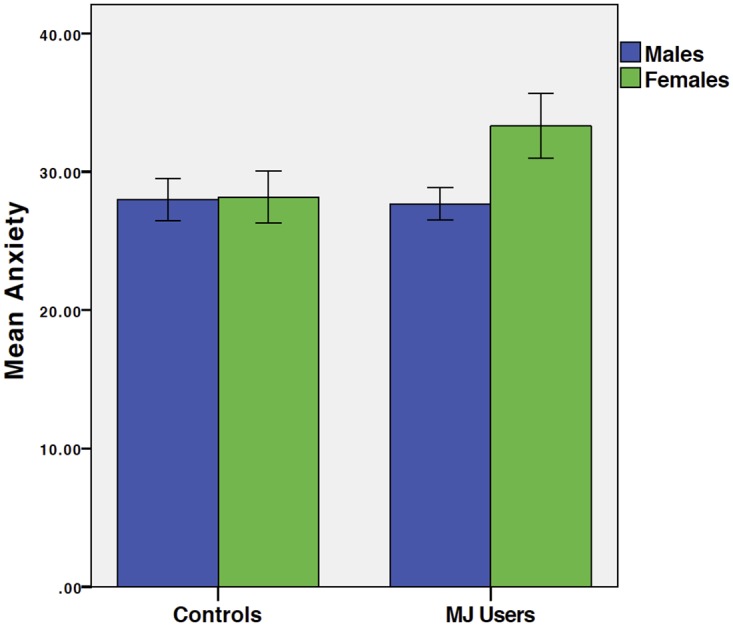
Anxiety by Group and Gender. Mean STAI State Anxiety raw score by group (left: controls; right: MJ users) and gender (blue: males; green: females). Results demonstrate that female MJ users have significantly higher anxiety than same-gendered controls and males.

**Fig 2:**
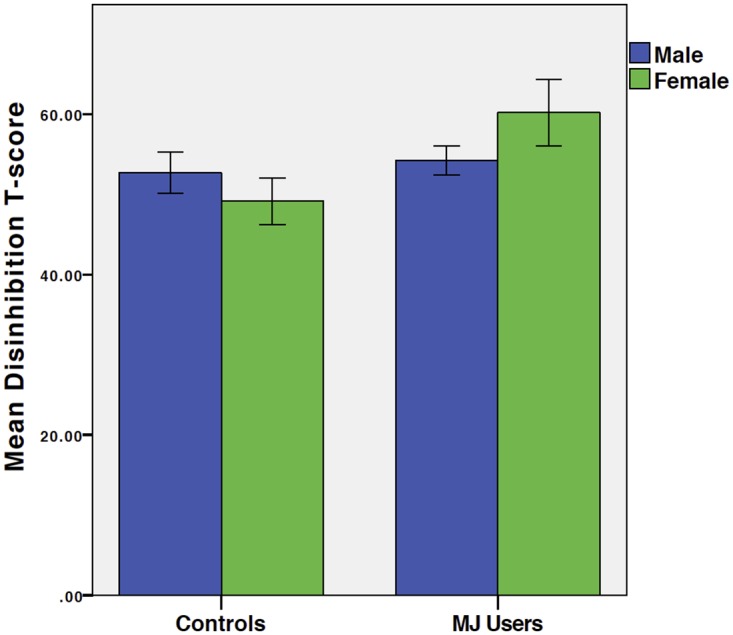
Disinhibition by Group and Gender. Mean FrSBe Disinhibition T-score by group (left: controls; right: MJ users) and gender (blue: males; green: females). Results demonstrate that female MJ users have significantly higher disinhibition than same-gendered controls and males.

MJ group*gender use predicted anxiety symptoms [beta =.22, t = 2.05, p =.044, f^2^ =.056]. Examination of marginal means revealed that MJ-using females demonstrated increased anxiety symptoms in comparison to males and same-gendered controls (see Fig 1). FrSBe **Disinhibition** was predicted by MJ*gender interactions [beta =.22, t = 2.07, p =.04, f^2^ =.11], with female users having greater disinhibition (see Fig 2).

#### Covariate Results. Cotinine Results

On the BIS/BAS, increased cotinine level predicted increased **drive** [beta =.27, t = 2.03, p =.046, f^2^ =.55], increased **reward responsiveness** [beta =.35, t = 2.70, p =.008, f^2^ =.053], and increased **BAS System Total** [beta =.33, t = 2.50, p =.02, f^2^ =.085]. Past year alcohol and ecstasy use did not significantly predict any results.

FrSBe **Apathy** and **Executive Dysfunction** were not significantly predicted by any measures.

## Discussion

The aims of the current study were to assess whether marijuana users demonstrated greater symptoms of anxiety, depression, and behavioral symptoms of executive dysfunction, while controlling for the effects of alcohol, cotinine, and ecstasy. Further, we sought to examine potential gender differences in these effects. Findings suggest that, after one week of abstinence, MJ users exhibited significantly greater depressive symptoms along with decreased fun-seeking, reward response, and Behavioral Approach Scale total scores. Gender significantly interacted with marijuana use in predicting anxiety and disinhibition, with females exhibiting higher levels of anxiety and disinhibition. Apathy and executive dysfunction were also not related to MJ group status, alcohol use, ecstasy use, and cotinine level often predicted an opposite pattern of BAS scores relative to the marijuana findings.

This study is consistent with prior findings regarding increased depressive symptoms in marijuana users [52, 72–77]. The eCB system is downregulated in response to repeated THC binding to CB1 [11], particularly in limbic regions[12], and the eCB system has been linked to increased mood symptoms [13–15], perhaps suggesting an underlying mechanism. In an independent sample, our group previously reported that increased marijuana use was predictive of increased depressive symptoms in young adults without independent Axis I mood disorders. Further, we found that frontolimbic poorer white matter integrity and volume were related to increased depressive symptoms and apathy in adolescent and young adult MJ users [25, 78]. Marijuana use may therefore lead to white matter abnormalities [25, 78], which in turn may lead to greater depressive symptoms. A recent meta-analysis has also reported that marijuana use increases the odds ratio of developing depression [74]. Importantly, the present study excluded for independent mood disorders, so these findings may not generalize to youth who develop depression prior to their onset of marijuana use. In addition, the effect size demonstrated was small, indicating that the relationship between marijuana use and depressive symptoms maybe subtle. More longitudinal studies are needed to assess the impact of marijuana use on the trajectory of mood symptoms and to establish causality.

Interestingly, the marijuana users in the present sample demonstrated decreased behavioral approach scores (BAS), a measure of response to rewarding events that is also thought to reflect aspects of impulsivity. Admittedly few studies have examined impulsivity in marijuana users with the BIS/BAS. One known study [51] found that increased BAS score was associated with lifetime experimentation, but not regular use, of marijuana, perhaps suggesting such appetitive desires are linked to novelty-seeking experimentation but not long-term behaviors; they further found increased BIS scores being related to transitioning into regular marijuana use, while our study found no relationship between marijuana and BIS total. In its initial validation, Carver and White [69] suggested that the BAS scale may not be reflective of impulsivity, per se, but of response to reward cues. Other research has suggested the BAS as a measure of positive or adaptive functioning [79]. Such decreased response to reward, fun seeking, and overall BAS, though at a small effect size in the present results, may therefore be more indicative of increased apathy and decreased mood, rather than impulsive traits. Indeed, mood disorders have previously been associated with decreased appetitive behaviors as measured by the BIS/BAS [80, 81]. Increased white matter integrity has also been positively correlated with BAS subscales in healthy adults [82]. Therefore, as our sample of marijuana users had greater depressive symptoms and as marijuana use has previously been linked to decreased white matter integrity and volume in frontolimbic regions [25, 34, 83], these findings may reflect more depressive symptomatology rather than classically defined impulsivity.

The present results indicate that female MJ users had increased anxiety and disinhibition compared to MJ-using males and non-using females. Females in general show higher rates of anxiety, although in this study only the MJ-using females demonstrated an elevation in anxiety. This is consistent with a previous study[59] has shown that females with cannabis use disorder have a higher incidence rate of mood and anxiety disorders, but these findings are the first to find these relationships in self-reported symptomatology. Brain regions involved with emotion identification demonstrate altered connectivity in young cannabis users [84, 85], which may account for difficulties identifying emotions and higher rates of experienced anxiety [86]. While gender findings are not always consistent, a growing body of literature suggests that female marijuana users may be more susceptible to marijuana-related emotional dysregulation. Females have previously been shown to exhibit greater executive dysfunction and depressive symptoms as predicted by aberrant prefrontal and amygdala volumes [39, 41]. Therefore, there is increasing evidence that females may be more susceptible to both underlying neurological deficits as well as anxiety and executive dysfunction deficits and should continue to be considered as a potentially important, though often subtle, factor in the neurocognitive effects of marijuana use.

Self-reported executive dysfunction was generally not statistically related to marijuana use and marijuana users did not differ significantly from controls in percentage of participants with elevations. This is in contrast to prior findings of increased self-reported executive dysfunction, apathy, and disinhibition predicting increased marijuana use [44, 45]. One reason for the lack of difference may be due to the fact that we controlled for alcohol use; while we did so in our group’s previous study [52], these inconsistent results may be due to the extent of combined use, or due to different characteristics of samples. Even so, the percentage of MJ users demonstrating clinical elevations is actually higher than our previous report [52]: apathy (previous study: 29%; current study: 35%), disinhibition (previous study: 18%; current study: 31%), and executive dysfunction (previous study: 12%; current study: 43%). Such elevations are consistent with previous studies of self-reported apathy in regular marijuana users and disinhibition as a predictor of later consumption of MJ in young adolescents [44, 87]. In the present study, the majority of participants did not meet clinical thresholds for anxiety and depression, even within the marijuana group, but given the immense cost of mental health burden [88], any elevations are of concern and highlight the need for prevention of both marijuana use and early intervention for general mental health issues in adolescents. Further, as Risher and colleagues[89] point out, as cognitive, and we would argue emotional, functioning are such key aspects of human functioning, a change of even 5% in these domains may be a red flag for further consideration. Given the relative elevations in symptomatology demonstrated by the present sample, findings look beyond pure statistical significance at even low levels of symptomatology as this may alter the lived experience of the individual. Particularly when considering the limitations of statistical significance [90], it is suggested that even subthreshold symptoms need to be considered as a potential issue in substance using adolescents and young adults.

The largely non-significant findings regarding the influence of alcohol use on psychiatric symptoms and executive dysfunction were in contrast with our hypotheses. Indeed, a number of studies have previously found heavy and binge drinking to be related to such deficits (see [55]). For example, Winward and colleagues [91] found that heavy episodic drinking adolescents had a range in executive function deficits in comparison to controls, such as in inhibition and cognitive switching. Others found that daily mood and anxiety fluctuations predict daily alcohol consumption in college students [92], and that mood and anxiety disorders increase the odds ratio of developing an alcohol use disorder [93]. Bekman and colleagues [94] found initial increased depressive and anxiety symptoms in recently abstinent heavy drinking adolescents, though affect improved after four-to-six weeks of monitored abstinence. However, given the known microstructural and neural changes that occur with alcohol use in adolescence ([95]; see[96]), perhaps the full deficits related to alcohol have not yet been manifested but, as Fleming and colleagues [97] suggest, a different mechanism has potentially been “locked-in” due to alcohol use during this vulnerable developmental time period, which may lead to larger deficits under the right circumstances in later life (such as in response to a significant stressor). It is also possible that there is a non-linear relationship such that light users may have fewer symptoms compared to non-users and heavy users. While not investigated here, future studies may need to address this.

Results suggest an opposite pattern of results found between marijuana use and cotinine levels in a measure of reward response and impulsivity, with marijuana use predicting decreased behavioral approach and cotinine level predicting increased behavioral approach. Given that the BAS subscales and total scales are measures of approach toward appetitive stimuli as well as theorized to be related to increased positive feelings [69], perhaps these results indicate recent use of tobacco products increases an individuals’ reward sensitivity and enjoyment of activities, at least on the short term. As marijuana users who co-use nicotine have been shown to have different brain-behavior relationships [98], greater consideration of the interactive influence of tobacco, in addition to alcohol as argued previously, is needed in future studies.

As with all studies, limitations should be noted. The sample size, while consistent with many studies in the literature, was nonetheless small, and should be replicated with more participants. This study was cross-sectional, and therefore more longitudinal studies are needed to determine causality. This is especially true given that potential bidirectional relationships exist, with marijuana use leading to increased symptomatology [74, 99], as well as some evidence for adolescent and young adult anxiety and mood symptoms leading to subsequent marijuana use [100, 101]. However, the exclusion of independent Axis I disorders prior to marijuana initiation or during marijuana abstinence suggests that these mood findings are not influenced by comorbid factors. This exclusion criteria may also underestimate the mood symptoms seen in comorbid marijuana and major depressive disorder patients, limiting the generalizability of the findings as other samples may demonstrate higher levels of marijuana use but have comorbid diagnoses. Clinically, MJ users may be demonstrating greater depressive symptoms, lowered drive, and greater apathy, and these factors need to be considered as potential treatment targets. MJ using participants may have been experiencing withdrawal symptoms that influenced their self-reported mood and executive functioning. However, seven days of abstinence was required, which is typically a long enough period of time for the most substantial withdrawal symptoms to subside and, in fact, mood does not appear to change significantly with withdrawal [64]. Finally, given the fact that the majority of participants were Caucasian, these results may not be generalizable to racial minorities; differences in mood and apathy in marijuana using minorities should be investigated in the future.

Future research is needed to understand the underlying mechanisms of the present findings. For example, greater understanding of underlying neurobiological mechanisms of frontolimbic functioning is needed, such as particular genotypes that may influence the endogenous endocannabinoid system (e.g., *FAAH*; [78]) or the downregulation of CB1 signaling [62]. As discussed previously, both marijuana and alcohol act on the endocannabinoid system and their effects may be altered by co-occurring use, and though the potential effect of co-occurring episodic use was not measured in the present study, future research should investigate the potential influence of simultaneous substance use.

In sum, the present study found behavioral deficits in mood, anxiety, and behavioral approach (drive, fun seeking, reward sensitivity) symptoms in marijuana users in comparison to healthy controls after a minimum of seven days of abstinence. We also found increased anxiety and disinhibition in female marijuana users. As no participants met criteria for an independent Axis I disorder, these findings suggest sub-clinical threshold deficits related to regular marijuana use provide additional evidence for increased prevention efforts in youth. Future research should assess potential methods of intervention that target frontolimbic function in young marijuana users.

## Supporting Information

S1 Data The supporting information file includes all data from the present analyses, as required by PLOSOne. (XLSX)
